# Supplementary material for: Microarchitectural Study of the Augmented Bone Following a Modified Ridge Splitting Technique: Histological and Micro-Computed Tomography Analyses
Source: J Clin Med. 2024 Nov 7;13(22):6679. doi: 10.3390/jcm13226679 (PMC11594336; doi:10.3390/jcm13226679)
Supplement: Supplementary file 1 [file jcm-13-06679-s001.zip › Supplementary Table S1.pdf]

microCT

test group=1, control group=2	BV/TV	BS/TV
1	8.1266586	0.02726014
1	25.40836106	0.0114898
1	16.75463742	0.01050023
1	25.56702559	0.0149533
1	12.71839612	0.01762259
1	21.99534213	0.01845444
1	15.42035547	0.02794997
1	24.84768271	0.02121901
1	32.82751817	0.01035681
1	31.96075449	0.01086258
1	33.45034529	0.03034902
1	12.53151998	0.02339722
1	27.43416903	0.00908018
1	20.19429143	0.01910768
2	21.02373901	0.01777713
2	13.89348148	0.0137894
2	6.20883734	0.00946402
2	13.53668218	0.01205138
2	16.36207439	0.01177071
2	15.37043545	0.01337775
2	10.69689158	0.01658377
2	10.56157332	0.02199142
2	9.73246232	0.02083627
2	12.10776227	0.01418851
2	11.54290207	0.01829978
2	7.99947165	0.02333073
2	7.93331639	0.01886658

Tb.Th	Tb.Sp	Tb.Pf	SMI
190.7719914	809.1434588	0.01192836	2.62545215
322.8755318	490.2459418	0.00029681	0.15499475
355.4053059	920.6985368	-0.00009608	-0.05490006
255.3778174	673.9734568	-0.00587379	-2.35685378
272.9395555	805.4328753	0.00664038	2.26086287
272.2072296	583.3606524	0.00686262	2.23120945
198.1988117	647.3736015	0.0099059	2.12649173
285.3035435	477.2273014	0.00466492	1.31907861
330.2158749	556.3481383	-0.00249661	-1.44636008
320.0253957	558.5442081	-0.00454398	-2.50988686
144.7249216	260.2563158	0.00878155	1.73611187
204.6675426	1167.017619	0.00429055	1.10027146
351.5185214	663.8656885	-0.00277239	-1.83193966
238.6499456	689.0495477	0.00421049	1.32213644
238.23262	591.1892184	0.00272375	0.91929868
330.486327	1258.592227	0.00621128	2.70263036
364.3251937	2099.91523	0.00087312	0.55353905
365.1797629	834.302287	0.00623261	3.10301917
296.6399543	820.5329439	0.00193873	0.98824728
293.4824861	837.4731822	0.00301334	1.35150316
257.4477038	875.7325409	0.00774972	2.80384681
164.5425042	1059.57151	0.00007297	0.01990756
213.4351196	900.2939917	0.00989646	2.84977896
296.9084925	842.0790452	0.00787546	3.33035348
252.1285315	880.0135432	0.00912747	2.99264866
239.6661206	892.3435738	0.01033737	2.65847662
227.1907673	981.2683229	0.00356809	1.13473431

Po.tot	Conn.
91.8733414	1573
74.59163894	156
83.24536258	405
74.43297441	4185
87.28160388	1248
78.00465787	2417
84.57964453	4029
75.15231729	5079
67.17248183	2175
68.03924551	2516
66.54965471	2573
87.46848002	2838
72.56583097	853
79.80570857	4278
78.97626099	2166
86.10651852	1533
93.79116266	222
86.46331782	378
83.63792561	880
84.62956455	978
89.30310842	459
89.43842668	2546
90.26753768	1213
87.89223773	354
88.45709793	1125
92.00052835	1139
92.06668361	529
