# Supplementary material for: Microarchitectural Study of the Augmented Bone Following a Modified Ridge Splitting Technique: Histological and Micro-Computed Tomography Analyses
Source: J Clin Med. 2024 Nov 7;13(22):6679. doi: 10.3390/jcm13226679 (PMC11594336; doi:10.3390/jcm13226679)
Supplement: Supplementary file 1 [file jcm-13-06679-s001.zip › Supplementary Table S2.pdf]

histomorphometry

test group=1, control group=2	BVP
1	0.298653222
1	0.441287571
1	0.589104654
1	0.490969518
1	0.475495163
1	0.508539355
1	0.505760021
1	0.518896034
1	0.675513079
1	0.304408156
1	0.574518738
1	0.38731951
1	0.422878001
2	0.341646107
2	0.339786125
2	0.600021606
2	0.239728741
2	0.534848212
2	0.391910318
2	0.399584537
2	0.363786131
2	0.520772439
2	0.370763864
2	0.548092182
2	0.473656506
